# Global research trends and hotspot evolution analysis of immunotherapy for allergic rhinitis: a multi-dimensional exploration based on bibliometrics

**DOI:** 10.1016/j.bjorl.2026.101798

**Published:** 2026-05-17

**Authors:** Jun Wu, Youwei Bao, Qi Chen, Xinhua Zhu

**Affiliations:** Nanchang University, Jiangxi Medical College, The Second Affiliated Hospital of Nanchang University, Department of Otorhinolaryngology Head and Neck Surgery, Nanchang, P.R. China

**Keywords:** Rhinitis, Allergic, Immunotherapy, Biological products, Bibliometrics, Precision medicine

## Abstract

**Objective:**

This study employs bibliometric methods to analyze global research trends and the evolution of hotspots in the field of Allergic Rhinitis (AR) immunotherapy between 2010 and 2024.

**Methods:**

A comprehensive search was conducted in the Web of Science Core Collection database for relevant literature published from 2010 to 2024. VOSviewer and CiteSpace were utilized to perform analyses on country/institutional collaboration, keyword co-occurrence and clustering, and citation bursts for the final set of 2470 included publications.

**Results:**

The annual global publication output demonstrated a steady growth trend. The United States, China, and major European countries emerged as the core research contributors. The evolution of research hotspots revealed a distinct “three-polar pattern”: a transition in treatment from traditional immunotherapy towards biologics, notably omalizumab and dupilumab; an upgrade in diagnosis from crude allergen extract testing to molecular component-resolved techniques; and the integration of guidelines, digital health, and patient-reported outcomes to form a closed-loop management system.

**Conclusion:**

This study identifies a comprehensive shift within the AR immunotherapy field towards precision medicine. Future efforts should focus on real-world evidence generation for biologics, the translation of biomarkers into clinical practice, and cost-effectiveness evaluations, aiming to establish a “precise diagnosis-targeted intervention-dynamic management” clinical pathway.

**Level of evidence:**

Level 1.

## Highlights

• First bibliometric analysis of global allergic rhinitis immunotherapy research.
• Triple shift: therapy→biologics, diagnostics→molecular, care→integrated.
• Anti-IgE (omalizumab) and anti-IL-4Rα (dupilumab) are key biologics.

## Introduction

Allergic Rhinitis (AR) is a highly prevalent chronic inflammatory disease worldwide, affecting the quality of life of 10%-40% of the global population, with its incidence continuing to rise. The condition not only causes typical symptoms such as nasal congestion, rhinorrhea, and sneezing but also has a comorbidity rate with asthma as high as 40%, imposing a substantial socioeconomic burden.^1^ Allergen Immunotherapy (AIT) is recognized as the only etiological treatment strategy capable of altering the natural course of AR, due to its ability to induce long-term antibody production in patients and suppress the allergic response process at the immune level.^2^, ^3^, ^4^

Over the past three decades, the field of AR immunotherapy has undergone revolutionary changes, evolving from traditional Subcutaneous Immunotherapy (SCIT) to Sublingual Immunotherapy (SLIT), and more recently, to the emergence of biologics and novel interventional techniques.^5^, ^6^, ^7^, ^8^ However, given the rapidly iterating technological approaches and intricate mechanistic research, there is a pressing need to systematically map the knowledge structure, evolution of research hotspots, and collaboration networks in this field. Traditional article reviews, reliant on subjective screening, struggle to quantify trends and reveal cross-disciplinary connections. Therefore, this study utilizes the Web of Science database and analytical tools such as VOSviewer, CiteSpace, and the R package bibliometrix to analyze global trends, key hotspots, and research trajectories in AR immunotherapy. The aim is to guide future research directions, address gaps in the understanding of immunotherapy's role in AR management, and provide a scientific basis for optimizing prevention and control strategies while advancing the translation of precision medicine.^9^, ^10^, ^11^

## Methods

### Data sources and retrieval strategies

#### Data source and search strategy

A systematic article search was conducted using the Web of Science Core Collection, including the Science Citation Index Expanded (SCI-Expanded) and the Social Sciences Citation Index (SSCI) databases. The search strategy was as follows: TS = (("allergic rhiniti" OR "hay fever" OR "seasonal allerg" OR "perennial allerg") AND ("immunotherap" OR "AIT" OR "desensitization" OR "allergen immunotherap" OR "SLIT" OR "sublingual immunotherap" OR "SCIT" OR "subcutaneous immunotherap" OR "intralymphatic immunotherap" OR "biologic agent" OR "monoclonal antibod" OR "dupilumab" OR "omalizumab" OR "mesenchymal stem cell*" OR "MSC therapy")).

### Article screening and inclusion criteria

**Fig. 1:**
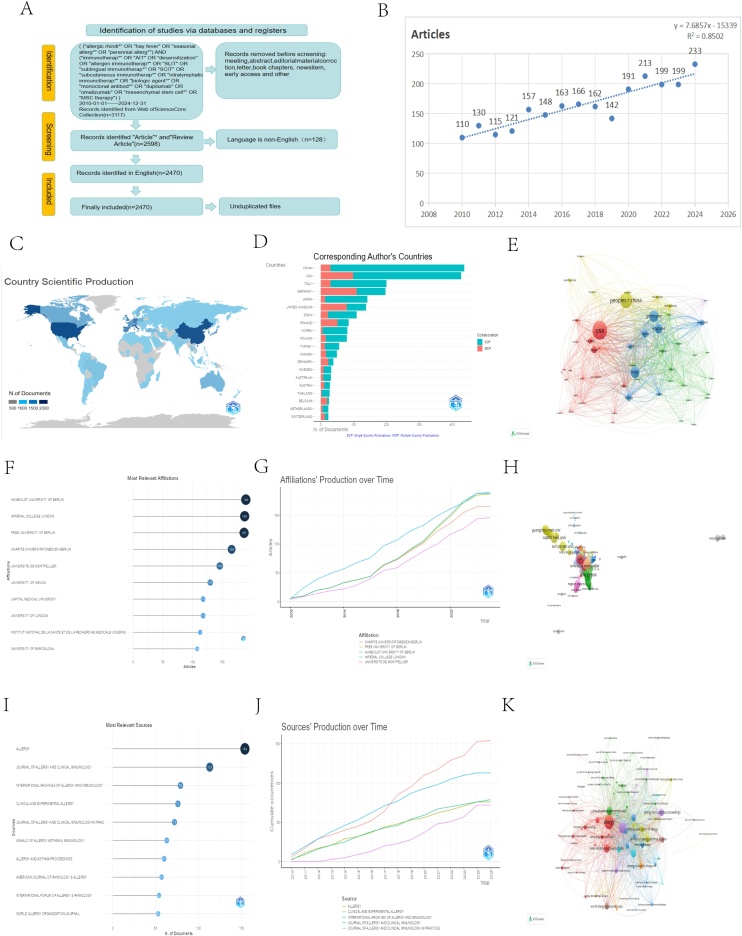
(A) Article search workflow for this study. (B) Annual publication trends. (C) Geographical distribution of corresponding authors by country (based on publication statistics). (D) Geographic distribution of the top 20 countries/regions with most publications by corresponding authors. (E) Country Cooperation Network. (F) Top 10 institutions by publication volume. (G) Annual publication volume of institutions. (H) Visualization of institutional collaboration networks. (I) Top 10 journals by publication volume. (J) Annual publication volume of journals. (K) Visualization of journal collaboration networks.

To minimize potential bias from routine database updates, all searches and data downloads were completed within a single day on 2025.10.20. The preliminary publication time frame was set from January 1, 2010, to December 31. The initial search yielded 3,117 publications. Following manual screening to remove irrelevant records, 2,598 publications from the last 15-years were retained. After further filtering by document type and removal of duplicates, 2,470 publications were ultimately included for analysis (for example, meeting summaries, editorials, letters, etc.). This final dataset comprised 1,778 original articles and 692 reviews, providing a robust foundation for subsequent bibliometric analysis (Fig. 1A).^12^

## Data analysis

Visualization network construction: VOSviewer 1.6.18 was used to generate national/institutional cooperation network and keyword co-occurrence clustering.

Evolution path analysis: CiteSpace 6.2.R4 was used for keyword emergence detection and time zone mapping. Impact evaluation: combined with the Citation frequency (Citation), H-index and the proportion of highly cited papers (Top 1%); Statistical methods: Pandas and Matplotlib libraries in Python 3.10 were used to complete the trend fitting and statistical test. All software and tools mentioned are publicly available versions, with no proprietary or restricted licenses utilized.

## Results

### Publication trend analysis

This study analyzed 2,470 publications on AR immunotherapy from 2010 to 2024, contributed by 9,311 researchers from 1,838 institutions across 83 countries. The annual publication output demonstrated a trend of steady expansion, with the number of articles increasing from 110 in 2010 to a peak of 233 in 2024 (Fig. 1B).

The distribution of publications by country, based on the affiliations of corresponding and first authors, highlighted the leading contributions of China (437 publications) and the United States (427 publications). Other high-output countries included Italy (200) and Germany (197) (Fig. 1C‒D). The international collaboration network further illustrates the central role these countries play in global research efforts (Fig. 1E).

### Core institutions and cooperation networks

The most prolific institutions were Humboldt University of Berlin, Free University of Berlin, and Imperial College London. These leading contributors also maintained a dense collaboration network, underscoring the role of inter-institutional partnerships in advancing this field. Humboldt University of Berlin was the most productive institution with 190 publications (Fig. 1F), followed by Imperial College London (188), Free University of Berlin (187), Charité – Universitätsmedizin Berlin (166), and Université de Montpellier (146). Within the collaboration network, frequent cross-regional partnerships among numerous institutions were observed, where a higher number of connections indicates a greater level of collaborative activity (Fig. 1F‒H).

### Distribution of high impact journals

The 2,470 analyzed publications were disseminated across 437 academic journals. The top 10 most productive journals collectively published 781 articles, accounting for 31.62% of the total output. Among these, Allergy (154 articles), the Journal of Allergy and Clinical Immunology (113), and the International Archives of Allergy and Immunology (79) emerged as the three most influential journals, underscoring their pivotal role in allergy research. Fig. 1I presents the annual publication trends for these key journals, while Fig. 1J displays their total citation counts. Furthermore, the journal co-citation network, illustrated in Fig. 1K, reveals the intellectual relationships and knowledge flow between these publications.

### Authors and co-cited authors

**Fig. 2:**
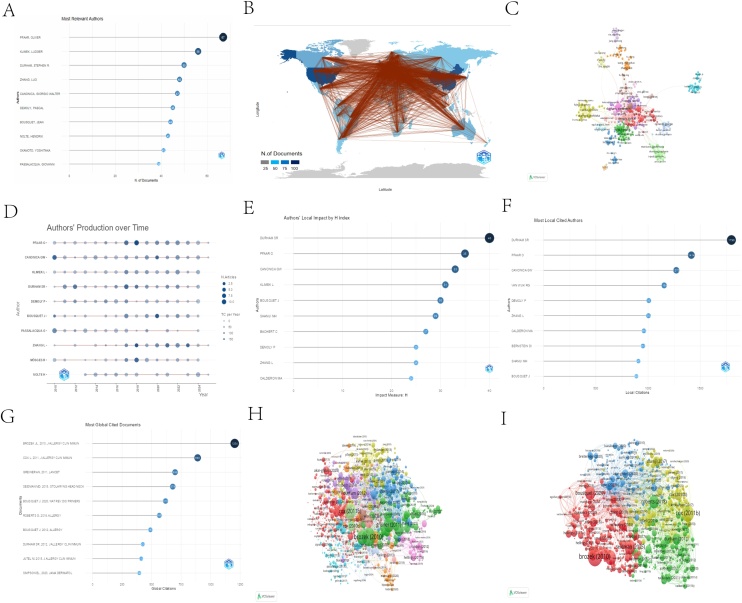
(A) Top 10 authors by publication volume. (B) Author collaboration network map. (C) Co-author network. (D) Annual publication details of top 10 authors. (E) Local citation H-index for authors. (F) Most Local Cited Authors. (G) Most Global Cited Documents. (H) Citation network relationships. (I) Citation network of the article.

Analysis of the author collaboration network identified the leading contributors. The most prolific author was Oliver Pfarr (67 publications), followed by Ludger Klimek (55 publications) and Stephen R. Durham (50 publications). Furthermore, the examination of co-authorship networks (Fig. 2B The red line indicates collaboration between authors), annual publication output, and H-index metrics collectively underscores their central influence and sustained productivity in AR immunotherapy research (Fig. 2A‒F).

### Article analysis

Analysis of the included article identified the most frequently cited works. The three most cited publications were Brozek JL et al. (2010, J Allergy Clin Immunol), Cox L et al. (2011, J Allergy Clin Immunol), and Greiner AN et al. (2011, Lancet), underscoring the influential role of these journals in the field. The study by Brozek JL et al. (2010) demonstrated the strongest impact, with a total of 1,204 citations, 75.25 citations per year, and a normalized citation count of 22.14. Fig. 2H presents the direct citation network of these publications, while Fig. 2I visualizes their co-citation relationships.

### Keywords co-occurrence and burst analysis

**Fig. 3:**
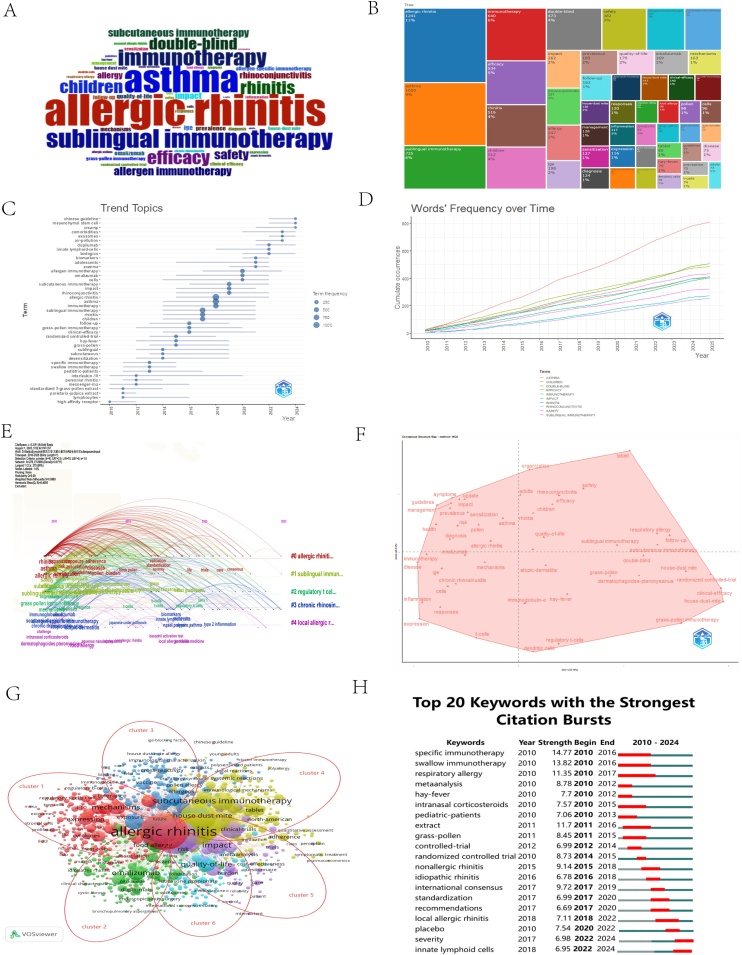
(A) Keyword Cloud. (B) Keyword Tree Graph. (C) Trend Topics. (D) Word's Frequency Over Time. (E) Timeline clustering analysis based on Citespace. (F) Factor Analysis Results. (G) Keyword Clustering Analysis Results. (H) Top 20 Keywords with the highest citation count.

Keyword co-occurrence analysis extracted 6,073 terms from the publications. The most frequent keywords were “allergic rhinitis” (1,241 occurrences), “asthma” (1,052), “subcutaneous immunotherapy” (726), “immunotherapy” (642), and “efficacy” (534) (Fig. 3A‒B). The thematic evolution map (Fig. 3C) illustrates a clear trajectory of research priorities from 2010 to 2024. Early studies focused on extending traditional Allergen Immunotherapy (AIT) and establishing its standardization and safety. The research paradigm has since evolved from basic mechanism exploration and clinical efficacy validation towards personalized treatment (for children/specific allergens), with current emphasis shifting to holistic disease management, encompassing quality of life and economic outcomes (Fig. 3A‒E).

Cluster analysis of keywords helped identify the field's major research themes. The six primary clusters, each representing a unique focus area, are as follows (Fig. 3G): Cluster 1 (Pharmacotherapy & Alternative Medicine): Encompasses drug treatments (antihistamines, biologics), desensitization therapies, and alternative treatments (acupuncture) for allergic diseases like asthma and atopic dermatitis, with a focus on disease control across age groups, particularly adolescents. Cluster 2 (Immunological Mechanisms): Focuses on the immunologic basis of allergic diseases, including immune cells (B-cells, basophils, dendritic cells), cytokines, signaling pathways, and disease mechanisms like the atopic march. Cluster 3 (Clinical AIT Trials): Centers on AIT, emphasizing its efficacy, safety, adherence, and cost-effectiveness in pediatric populations, primarily through methodologies like randomized controlled trials. Cluster 4 (Molecular Allergology & Diagnostics): Delves into the molecular characteristics of specific allergens (pollen, dust mites, food allergens), cross-reactivity, diagnostic techniques (component-resolved diagnostics), and environmental factors (climate change). Cluster 5 (Guidelines & Integrated Management): Integrates international guidelines and consensus (ARIA, EAACI), long-term disease management strategies, health economics, patient-reported outcomes, and emerging digital health technologies (mHealth) for whole-life-cycle care. Cluster 6 (Patient Outcomes & Health Economics): Focuses on quality of life, specific treatment effects, and costs, considering the balance between healthcare burden and public health.

Keyword burst detection further revealed the field's evolving trends (Fig. 3H). The top 20 keywords with the strongest citation bursts are shown. From 2010 to 2016, “specific immunotherapy” had the highest burst strength (14.77), followed by “sublingual immunotherapy” (13.82) and “respiratory allergy” (11.35). Notably, bursts for “innate lymphoid cells” and “severity” have persisted until the present, reflecting the latest research frontiers.

## Discussion

This bibliometric analysis of AR immunotherapy research from 2004 to 2024 reveals a significant expansion in research output and a distinct evolution of hotspots. The United States, China, and European nations constituted the core driving forces behind this global endeavor, collectively propelling a paradigm shift from traditional therapies towards precision medicine.^1^^,^^2^^,^^9^^,^^13^ At the national and institutional levels, the United States maintained a leading position in frontier exploration, characterized by its high publication output and extensive international collaborative network. China followed closely, demonstrating rapid growth in research volume, with institutions like Capital Medical University making notable contributions. Europe, with Germany and Italy at its center, played a pivotal role in basic immunology and multi-center clinical trials, facilitated by the tightly-knit collaborative networks of institutions such as Humboldt University of Berlin and Imperial College London.

Journal analysis identified Allergy and the Journal of Allergy and Clinical Immunology as the most influential knowledge-dissemination platforms in the field. These journals not only led in publication volume but also served as the primary venues for publishing highly-cited guidelines and groundbreaking clinical trials.^14^^,^^15^ The evolution of their published content directly mirrors the discipline's shift in focus from the standardization of immunotherapy towards the efficacy of biologics and personalized strategies. Concurrently, journals such as International Archives of Allergy and Immunology have played a critical bridging role by connecting fundamental immunological mechanisms with clinical translation.^14^, ^15^, ^16^, ^17^, ^18^, ^19^, ^20^, ^21^

The most influential scholars in this field, such as Oliver Pfaar, Ludger Klimek, and Stephen R. Durham, have established the foundation for AR immunotherapy and modern biologic therapies through their seminal work.^22^^,^^24^ Their research, spanning large-scale clinical trials to the dissection of immune mechanisms, has not only shaped the current treatment paradigm but has also charted a course for the future development of targeted therapeutics.^22^, ^23^, ^24^

Co-occurrence and burst analysis of keywords visually delineated the evolution of research hotspots. The persistent high frequency of keywords such as “allergic rhinitis”, “asthma”, “quality of life”, and “efficacy” underscores the long-standing focus on AR as a systemic disease and its impact on patients. In recent years, the explosive growth of keywords like “biologics”, “dupilumab”, and “omalizumab” signifies a decisive shift in research focus towards targeted therapy.^25^, ^26^, ^27^, ^28^, ^29^ Concurrently, the emergence of “innate lymphoid cells” and “severity” indicates a growing research direction aimed at precision diagnosis and understanding specific lymphocyte-driven immune mechanisms. Although the keyword strength of traditional “subcutaneous immunotherapy” and “sublingual immunotherapy” has diminished, their continued co-occurrence with terms like “safety” and “children” confirms that their optimization and safety assessment remain vital components of research.

Notably, the research focus has shifted from popularizing therapies to achieving precision breakthroughs. In the early period (2010–2016), the keywords with the highest burst strength were “specific immunotherapy” (14.77) and “sublingual immunotherapy” (13.82), reflecting the standardized exploration of traditional AIT. In contrast, the recent rise of “dupilumab/omalizumab” (Cluster 1) and the persistent emergence of “innate lymphoid cells” (Fig. 3H) signify that biologics and immune mechanism research have become the new engine of the field. However, although the US and China lead in production volume, the density of institutional collaborations within these countries is weaker than in Europe (Fig. 1E), suggesting that regional synergistic potential has not yet been fully unleashed.^8^^,^^30^

In summary, research on AR immunotherapy is undergoing a profound transformation. Future research efforts should be dedicated to the following key directions: First, more real-world studies are needed to evaluate the long-term safety, efficacy, and cost-effectiveness of biologics, particularly in special populations such as children and patients with multiple comorbidities.^31^, ^32^, ^33^ Second, discoveries in basic immunology should be translated into clinical applications by exploring biomarkers that can predict treatment response, thereby enabling truly personalized therapy. Finally, there is an urgent need to integrate molecular diagnostics, targeted drugs, and digital health tools to establish a closed-loop management paradigm of “precise diagnosis-targeted intervention-dynamic monitoring”, alongside strengthening global research collaboration to collectively address AR as a worldwide health challenge.

This study provides a comprehensive analysis of the AR immunotherapy landscape from 2010 to 2024; however, several limitations should be acknowledged. First, the reliance on a single database, Web of Science, may result in the omission of relevant studies published in non-English languages or regional journals (from Latin America or Africa). Second, the keyword clustering analysis is constrained by the sensitivity of the algorithm (VOSviewer resolution parameter = 1.0), which may have led to the merging of some nuanced, subtopics. Third, our bibliometric approach did not integrate findings from animal models with clinical data, such as cross-species validation of microbiome interventions, which warrants future investigation through multi-omics integrated analysis. Future studies could address these limitations by expanding the article search to include databases such as Scopus and PubMed, and by fostering interdisciplinary collaborations with, for instance, public health agencies. Notwithstanding these limitations, the extensive cross-disciplinary coverage and robust citation-tracking capabilities of the Web of Science database ensure a high degree of accuracy and efficiency for this type of medical research analysis, justifying its use as the primary data source.

## Conclusion

Based on a bibliometric analysis of 2,470 publications from the Web of Science database (2010–2024), this study systematically elucidates global research trends and the evolution of hotspots in the field of AR immunotherapy. The core findings can be summarized as a “tripartite paradigm shift”. Therapeutically, the focus has moved from traditional SCIT/SLIT towards targeted biologics, notably omalizumab and dupilumab. Diagnostically, practices have advanced from the use of crude allergen extracts to molecular component-resolved techniques. In terms of disease management, the approach has evolved from isolated interventions to an integrated, full-cycle, closed-loop model that incorporates international guidelines (ARIA), digital health (mHealth), and patient-reported outcomes. Establishing and refining a “precise diagnosis-targeted intervention-dynamic management” framework is the essential pathway to advance AR care from symptomatic control towards disease modification.

## ORCID ID

Qi Chen: 0009-0001-8207-5406

Xinhua Zhu: 0000-0002-3776-0287

## Funding

This research received no specific grant from funding agencies in public, commercial, or not-for-profit sectors.

## Data availability statement

The authors declare that all data are available in repository.

## Declaration of competing interest

The authors declare no conflicts of interest.

## References

[1] Wheatley L.M., Togias A. (2015). Clinical practice. Allergic rhinitis. New Engl J Med..

[2] Pedregal-Mallo D., Pacheco E., Rodrigo J.P., Llorente J.L., Alvarez-Marcos C. (2020). Impact of immunotherapy on quality of life in patients with house dust mite allergic rhinitis. Allergy..

[3] Lou H., Huang Y., Ouyang Y. (2020). Artemisia annua-sublingual immunotherapy for seasonal allergic rhinitis: a randomized controlled trial. Allergy..

[4] Gao Y., Lin X., Ma J., Wei X., Wang Q., Wang M. (2020). Enhanced efficacy of dust mite sublingual immunotherapy in low-response allergic rhinitis patients after dose increment at 6 months: a prospective study. Int Arch Allergy Imm..

[5] Feng M., Zeng X., Su Q. (2020). Allergen immunotherapy-induced immunoglobulin G4 reduces basophil activation in house dust mite-allergic asthma patients. Front Cell Dev Biol..

[6] Wang W., Yin J., Wang X. (2020). Relationship between serum inhibitory activity for IgE and efficacy of Artemisia pollen subcutaneous immunotherapy for allergic rhinitis: a preliminary self-controlled study. Allergy Asthma Clin Immunol..

[7] Shen Z., Tan G., Zhong Z., Ding S., Wang F. (2019). Interactive network platform improves compliance and efficacy of subcutaneous immunotherapy for patients with allergic rhinitis. Patient Prefer Adherence.

[8] Jutel M., Brüggenjürgen B., Richter H., Vogelberg C. (2020). Real-world evidence of subcutaneous allergoid immunotherapy in house dust mite-induced allergic rhinitis and asthma. Allergy..

[9] Li H., Chen S., Cheng L. (2019). Chinese guideline on sublingual immunotherapy for allergic rhinitis and asthma. J Thorac Dis..

[10] Pechsrichuang P., Jacquet A. (2020). Molecular approaches to allergen-specific immunotherapy: are we so far from clinical implementation?. Clin Exp Allergy..

[11] Lam H.Y., Tergaonkar V., Ahn K.S. (2020). Mechanisms of allergen-specific immunotherapy for allergic rhinitis and food allergies. Bioscience Rep..

[12] Matricardi P.M., Potapova E., Forchert L., Dramburg S., Tripodi S. (2020). Digital allergology: towards a clinical decision support system for allergen immunotherapy. Pediat Allergy Immunol..

[13] Klimek L., Bachert C., Pfaar O. (2019). ARIA guideline 2019: treatment of allergic rhinitis in the German health system. Allergol Select..

[14] Nair P., Radford K., Nunomura S., Mukherjee M., Izuhara K. (2024). Response of sputum periostin to anti-T2 biologics treatment in severe asthma. Allergy..

[15] Ye X., Li Y., Fang B. (2023). Type 17 mucosal-associated invariant T cells contribute to neutrophilic inflammation in patients with nasal polyps. J Allergy Clin Immunol..

[16] Yıldız E., Çölkesen F., Arslan S., Evcen R., Sadi Aykan F., Kılınç M. (2023). Allergic diseases as a clinical phenotype marker in patients with common variable immunodeficiency. Int Arch Allergy Imm..

[17] Sio Y.Y., Gan W.L., Ng W.S. (2023). The ERBB2 exonic variant Pro1170Ala modulates mitogen-activated protein kinase signaling cascades and associates with allergic asthma. Int Arch Allergy Immunol..

[18] Guo C.L., Lu R.Y., Wang C.S. (2023). Identification of inflammatory endotypes by clinical characteristics and nasal secretion biomarkers in chronic rhinosinusitis with nasal polyps. Int arch allergy imm.

[19] Buchheit K.M., Shaw D., Chupp G. (2024). Interleukin-5 as a pleiotropic cytokine orchestrating airway type 2 inflammation: effects on and beyond eosinophils. Allergy..

[20] Byrwa-Hill B.M., Morphew T.L., Presto A.A., Fabisiak J.P., Wenzel S.E. (2023). Living in environmental justice areas worsens asthma severity and control: differential interactions with disease duration, age at onset, and pollution. J Allergy Clin Immunol..

[21] Siddiqui S., Wenzel S.E., Bozik M.E. (2023). Safety and Efficacy of Dexpramipexole in Eosinophilic Asthma (EXHALE): a randomized controlled trial. J Allergy Clin Immunol..

[22] Durham S.R., Shamji M.H. (2023). Allergen immunotherapy: past, present and future. Nat Rev Immunol..

[23] Bousquet J., Jutel M., Akdis C.A. (2021). ARIA-EAACI statement on asthma and COVID-19 (June 2, 2020). Allergy..

[24] Sousa-Pinto B., Jácome C., Pereira A.M. (2023). Development and validation of an electronic daily control score for asthma (e-DASTHMA): a real-world direct patient data study. Lancet Digit Health.

[25] Agache I., Song Y., Posso M. (2021). Efficacy and safety of dupilumab for moderate-to-severe atopic dermatitis: a systematic review for the EAACI biologicals guidelines. Allergy..

[26] Agache I., Rocha C., Beltran J. (2020). Efficacy and safety of treatment with biologicals (benralizumab, dupilumab and omalizumab) for severe allergic asthma: a systematic review for the EAACI Guidelines - recommendations on the use of biologicals in severe asthma. Allergy..

[27] Agache I., Song Y., Alonso-Coello P. (2021). Efficacy and safety of treatment with biologicals for severe chronic rhinosinusitis with nasal polyps: a systematic review for the EAACI guidelines. Allergy..

[28] Agache I., Song Y., Rocha C. (2020). Efficacy and safety of treatment with dupilumab for severe asthma: a systematic review of the EAACI guidelines-Recommendations on the use of biologicals in severe asthma. Allergy..

[29] Atkinson C., Burbank A., Schworer S., Hernandez M. (2022). Omalizumab treatment is associated with improvement in outcomes in children with asthma despite pretreatment serum IgE exceeding dosing guidelines. J Allergy Clin Immunol..

[30] Gotua M., Gamkrelidze A., Rukhadze M. (2019). 2020 Aria care pathways for allergic rhinitis - Georgia. Georgian Med News..

[31] Soriano V.X., Ciciulla D., Gell G. (2023). Complementary and allergenic food introduction in infants: an umbrella review. Pediatrics..

[32] Caillaud D., Toloba Y., Raobison R. (2014). Health impact of exposure to pollens: a review of epidemiological studies. Rev Mal Respir..

[33] Bellanti J.A., Settipane R.A. (2019). Innate lymphoid cells: a new family of lymphocytes with involvement from worms to allergic disease. Allergy Asthma Proc..

